# MicroRNA Profiling and Bioinformatics Target Analysis in Dorsal Hippocampus of Chronically Stressed Rats: Relevance to Depression Pathophysiology

**DOI:** 10.3389/fnmol.2018.00251

**Published:** 2018-08-06

**Authors:** Mauricio Muñoz-Llanos, María A. García-Pérez, Xiaojiang Xu, Macarena Tejos-Bravo, Elena A. Vidal, Tomás C. Moyano, Rodrigo A. Gutiérrez, Felipe I. Aguayo, Aníbal Pacheco, Gonzalo García-Rojo, Esteban Aliaga, Paulina S. Rojas, John A. Cidlowski, Jenny L. Fiedler

**Affiliations:** ^1^Laboratory of Neuroplasticity and Neurogenetics, Faculty of Chemical and Pharmaceutical Sciences, Department of Biochemistry and Molecular Biology, Universidad de Chile, Santiago, Chile; ^2^National Institute of Environmental Health Sciences, National Institutes of Health, Department of Health and Human Services, Durham, NC, United States; ^3^Centro de Genómica y Bioinformática, Facultad de Ciencias, Universidad Mayor, Santiago, Chile; ^4^Millennium Institute for Integrative Biology (iBio), FONDAP Center for Genome Regulation, Departamento de Genética Molecular y Microbiología, Pontificia Universidad Católica de Chile, Santiago, Chile; ^5^Department of Kinesiology, Faculty of Health Sciences, Universidad Católica del Maule, Talca, Chile; ^6^Escuela de Química y Farmacia, Facultad de Medicina, Universidad Andres Bello, Santiago, Chile

**Keywords:** restraint stress, dorsal hippocampus, miRNA, neuroplasticity, mood disorder

## Abstract

Studies conducted in rodents subjected to chronic stress and some observations in humans after psychosocial stress, have allowed to establish a link between stress and the susceptibility to many complex diseases, including mood disorders. The studies in rodents have revealed that chronic exposure to stress negatively affects synaptic plasticity by triggering changes in the production of trophic factors, subunit levels of glutamate ionotropic receptors, neuron morphology, and neurogenesis in the adult hippocampus. These modifications may account for the impairment in learning and memory processes observed in chronically stressed animals. It is plausible then, that stress modifies the interplay between signal transduction cascades and gene expression regulation in the hippocampus, therefore leading to altered neuroplasticity and functioning of neural circuits. Considering that miRNAs play an important role in post-transcriptional-regulation of gene expression and participate in several hippocampus-dependent functions; we evaluated the consequences of chronic stress on the expression of miRNAs in dorsal (anterior) portion of the hippocampus, which participates in memory formation in rodents. Here, we show that male rats exposed to daily restraint stress (2.5 h/day) during 7 and 14 days display a differential profile of miRNA levels in dorsal hippocampus and remarkably, we found that some of these miRNAs belong to the miR-379-410 cluster. We confirmed a rise in miR-92a and miR-485 levels after 14 days of stress by qPCR, an effect that was not mimicked by chronic administration of corticosterone (14 days). Our *in silico* study identified the top-10 biological functions influenced by miR-92a, nine of which were shared with miR-485: Nervous system development and function, Tissue development, Behavior, Embryonic development, Organ development, Organismal development, Organismal survival, Tissue morphology, and Organ morphology. Furthermore, our *in silico* study provided a landscape of potential miRNA-92a and miR-485 targets, along with relevant canonical pathways related to axonal guidance signaling and cAMP signaling, which may influence the functioning of several neuroplastic substrates in dorsal hippocampus. Additionally, the combined effect of miR-92a and miR-485 on transcription factors, along with histone-modifying enzymes, may have a functional relevance by producing changes in gene regulatory networks that modify the neuroplastic capacity of the adult dorsal hippocampus under stress.

## Introduction

Neuroplasticity is considered a continuous process that permits short- to long-term brain remodeling in response to an experience and changing environment (McEwen and Gianaros, 2010). This process involves several mechanisms, ranging from synaptic remodeling to functional modification of synapses and neural circuitries (McEwen and Gianaros, 2010). In general, any acute threat to an individual, either physical or emotional in nature, triggers the activation of the stress system, including the sympathetic nervous system and the hypothalamic–pituitary–adrenal (HPA) axis and release of adrenal glucocorticoids, which are all responses that allow the organism to adapt (Chrousos and Harris, 1998; McEwen and Gianaros, 2010). Nonetheless, prolonged stress response may lead to a maladaptive response by favoring the onset or exacerbation of several stress-related disorders, including major depressive disorder (MDD) (Pittenger and Duman, 2008; Fernandez-Guasti et al., 2012). Depressed subjects show hippocampal volume reduction (Malykhin and Coupland, 2015) and cognitive deficits associated with altered hippocampal and prefrontal cortex functioning, along with a reduced complexity of dendritic trees; nonetheless, little is known about the mechanisms involved (Pittenger and Duman, 2008). One hypothesis considers that exposure to stressors alters neuroplasticity processes required for the maintenance and reestablishment of neuro-circuitry functioning (Pittenger and Duman, 2008).

Studies conducted in rodents under chronic stress models that recapitulate several symptoms of MDD have revealed changes in several forms of hippocampal neuroplasticity (Kim et al., 2015). Chronically stressed rodents show several cyto-architecture modifications in the hippocampus, including dendritic arbor simplification (Pinto et al., 2015) and dendritic spine loss in CA1 pyramidal neurons (Castaneda et al., 2015; Garcia-Rojo et al., 2017), changes that have been linked to reduced expression of the brain derived neurotrophic factor (BDNF) (Smith et al., 1995). Furthermore, chronic stress impairs hippocampus-dependent memory (Conrad et al., 1996), which can be associated with changes in the two forms of synaptic efficacy; i.e., long-term potentiation (LTP) and long-term depression (LTD) (Kim et al., 2015). All of these stress-induced neuroplasticity modifications in rodents can explain the impairment of learning and memory associated with hippocampal functioning (Conrad et al., 1996), which may be related to stress-related changes in gene expression.

Significant research has unveiled that microRNAs (miRNAs) mediate post-transcriptional gene silencing, playing a crucial role in brain development, synaptic plasticity, and neuropathology (Aksoy-Aksel et al., 2014). Emerging evidence recently reviewed has shown that miRNA expression can be differentially modulated by the extent of stress exposure (Hollins and Cairns, 2016). For instance, acute stress triggers variations in a higher number of miRNAs compared to subchronic stress in the prefrontal cortex of mice (Rinaldi et al., 2010). Moreover, evidences have also shown that acute stress produces the opposite effect when contrasted to chronic stress (Meerson et al., 2010). Some studies have evaluated the effect of chronic stress on the expression of miRNAs in the hippocampus. For instance, chronic restraint stress in male rats increases the expression of miR-138 (Castaneda et al., 2015). At least *in vitro*, this miRNA reduces the size of dendritic spines in cultured hippocampal neurons (Siegel et al., 2009), an effect that may be linked to the reduction in spine density observed in CA1 pyramidal neurons of chronically stressed rats (Castaneda et al., 2015). Chronic unpredictable mild stress (CUMS) triggers a rise in miR-182 levels at the hippocampus in rats and its overexpression exacerbates stress-induced depression-like behavior, a modification associated with a reduction in the levels of BDNF and cAMP responsive element binding protein 1 (CREB1) transcription factor (Li et al., 2016). Another study using microarray chip analyses and subsequent confirmation by RT-qPCR revealed that 35 days of CUMS mainly up-regulates a few miRNAs (miR-382-3p, miR-183-5p, miR-3573-5p, miR-202-3p, miR-493-3p) in male rats (Zhou et al., 2017). The miRNA target prediction and functional analysis conducted in this study revealed enrichment in numerous gene ontology terms and signaling related with depressive disorder (Zhou et al., 2017). However, in this study, authors used left hippocampal tissues after behavioral testing (Zhou et al., 2017) that may produce some biases in the study.

To date there is no study evaluating whether stress exposure chronicity (defined as days of stress exposure) determines a profile of miRNA expression, especially in dorsal hippocampus, an area that has a pivotal role in learning and memory in rodents (Fanselow and Dong, 2010). This portion of the hippocampus has called our attention because it is particularly sensitive to chronic stress, displaying an impairment of LTP induction (Miller et al., 2018) and reduction of dendritic spine density (Castaneda et al., 2015; Garcia-Rojo et al., 2017), reductions in NR1 and NR2A NMDA receptor subunit levels (Pacheco et al., 2017) and BDNF expression (Bravo et al., 2009); changes that altogether, indicate dorsal hippocampus dysfunction. Considering all these antecedents, we hypothesized that stress chronicity exposure determines a particular profile of miRNA expression in dorsal hippocampus that may influence several aspects of neuroplasticity in this structure.

By microarray analyses, the present study evaluates the effect of stress chronicity exposure on: physiological stress-related markers and miRNA expression profile in rat dorsal hippocampus, and by gene prediction and functional annotation analysis, we propose how these miRNAs may influence neuroplasticity mechanisms in the hippocampus.

## Materials and Methods

### Animals

Adult male Sprague-Dawley rats maintained at the Faculty of Chemical and Pharmaceutical Sciences, Universidad de Chile, were used in this study. Efforts were made to reduce both the number of animals used and their suffering. The rats were handled in compliance with the National Institutes of Health Guide for Care and Use of Laboratory Animals (NIH Publication, eighth Edition, 2011) under an experimental protocol approved by the Ethical Committee of the Faculty of Chemical and Pharmaceutical Sciences, Universidad de Chile (CBE2011-7-4), and the Science and Technology National Commission (CONICYT). Two-month-old Sprague Dawley rats (320–350 g) were given free access to water and pelleted food and were maintained at 22°C with humidity of 55% and photoperiod cycle of 12 h (lights on from 7:00 am to 7:00 pm).

### Chronic Stress Procedure and Tissue Collection

Prior to the experimental procedures, rats were handled once per day for a 1-week period, during the light phase of the photoperiod cycle. In the case of the stress experimental group, the rats were housed in a group of three to four rats and maintained in the experimental room. The stress procedure was conducted between 9:00 am and 12:00, as previously reported (Pacheco et al., 2017). In brief, the rats were introduced in transparent acrylic tubes (25 cm × 8 cm) during 2.5 h during 7 days (*n* = 9) or 14 days (*n* = 17). During the restraint, animals were placed in a cage in groups of three to four rats and the fecal pellet output during the stress session was evaluated. After this procedure, the stressed group of animals was placed in the home cage with new bedding. On the other hand, and considering that animal isolation may trigger a stress response, control animals (*n* = 16) were kept in the groups of three to four rats in the animal room located away from the experimental room. In order to determine the fecal output in control group, the bedding was changed each morning and after 2.5 h, the cage was inspected for fecal output quantification every day during 14 consecutive days. Hence, the estimation of fecal output corresponds to the mean value for a control group.

**FIGURE 1:**
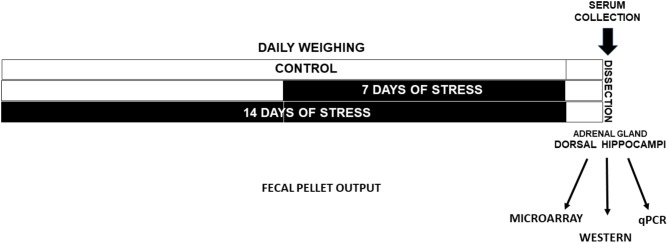
Experimental design. Adult male rats were weighed every day in the morning. One group was stressed during 14 days (Stress-14d) and after 7 days other was stressed during 7 days (Stress-7d). Fecal output during the restraint stress procedure (2.5 h/day) was registered. Twenty four hours after the last treatment, animals were decapitated and a blood sample was taken to determine serum corticosterone levels. Adrenal glands were removed and weighed, and the dorsal hippocampi were dissected and rapidly frozen in liquid N_2_.

Rats were decapitated rapidly 24 h after the last stress exposure (between 9:00 am and 12:00 am) and trunk blood was collected for corticosterone (CORT) determination in serum, as previously described (Pacheco et al., 2017) (**Figure 1**). The dorsal hippocampi relative to bregma −3.10 to −4.44 mm coordinates (Paxinos, 1982) were rapidly dissected at 4°C and frozen under liquid N_2_ and kept at −80°C until processing for RNA isolation and protein extract. Adrenal glands were rapidly dissected and weighed (**Figure 1**). We conducted two experimental series groups in which the first series was used for the microarrays, and the second one to: (i) validate the miRNAs, (ii) quantify some mRNAs by qPCR, and (iii) quantify protein levels by western blot analysis.

### Corticosterone Administration

Hormone administration was conducted as previously described (Ulloa et al., 2010). In brief, rats were injected s.c. once per day with a 30 mg/kg/day dose of CORT (Sigma-Aldrich, St. Louis, MO, United States) suspended in propylene glycol (CORT group, *n* = 5) every day for 14 days and control animals (*n* = 5) were injected with a similar volume of vehicle. Rats were decapitated 24 h after the last CORT administration and a blood sample was collected for hormone determination as we have previously described (Pacheco et al., 2017).

### RNA Isolation

Purified RNAs from dorsal hippocampi of the rat were isolated as we have previously described (Castaneda et al., 2015) by using the RNeasy Mini Kit (^1^QIAGEN, Hilden, Germany), that allows the separation of RNAs according to their size (RNAs < 200 nt and >200 nt). RNA concentration and purity were determined at OD260/280 and samples with an absorbance ratio between 1.8 and 2.0 were chosen. RNA integrity was evaluated by nondenaturing agarose gel electrophoresis.

### Chip-Based miRNA Expression Analysis and Target Genes Network Analysis

Analysis of miRNA expression was conducted using Affymetrix GeneChip^TM^ Version 3 miRNA arrays (680 mature rat and 486 rat pre-miRNA probe sets), following the Affymetrix hybridization protocols. Purified RNAs (<200 nt) were labeled using the Affymetrix FlashTag^TM^ Biotin HSR kit, according to the manufacturer’s protocol. Each sample was then hybridized under standardized conditions (16 h at 48°C) in a hybridization oven (Affymetrix) and array slides were stained with streptavidin/phycoerythrin and washed according to the manufacturer’s protocol. Finally, arrays were scanned using the Affymetrix GCS 3000 7G and GeneChip^®^ Operating Software (AGCC; Version 3.2). The microarray data are available in the Gene Expression Omnibus repository at the National Center for Biotechnology Information with the accession number GSE117046.^2^

To further analyze the microarray datasets, two tools were utilized: (1) Differential Expression Analysis using LIMMA-Bioconductor with R software^3^ and (2) the Analysis in Partek^®^ Genomics Suite^TM^ (Partek, Inc., St. Louis, MO, United States). For both tools, a single log scale normalized expression measure was obtained for each probe set after background correction and normalization between samples. In order to evaluate the quality of the microarray data and to identify any unusual data in the array slides, several plots (Box plots, density plots, MA plots) were generated for both raw and normalized data. For the method based in R software, the statistical significance *P*-value of the log ratio for each probe was determined by a modification of the standard *t*-test by using an empirical Bayesian approach. For Partek, ANOVA was used to identify differentially expressed probes. Probes that had *P* < 0.05 were considered to be differentially expressed between experimental groups and controls. Among the significant probes, only rat miRNA probes were kept. To further refine the selection of significant genes, common significant genes found with the two biostatistical tools were used for downstream analysis. Heat maps were generated using BioConductor package HeatPlus. Dendrograms of samples (columns) and genes (row) were generated by hierarchical clustering. Color scale was from threefold lower (log2-fold = −1.585) than the mean (blue) to threefold higher (log2-fold = 1.585) than the mean (red).

Validated miRNAs were further analyzed for their mRNA targets using the Ingenuity Pathway Analysis (IPA) tool (version 21249400) (Ingenuity Systems, Redwood City, CA, United States), as previously described (Kadmiel et al., 2016). The IPA tool allows to access both experimentally validated and predicted mRNA targets from TargetScan and TarBase. Enrichment or overlapping was determined by IPA using Fisher’s exact test (*P* < 0.05). The target genes were further analyzed with IPA core analysis module for functional enrichment of target genes to understand their putative impact in several biological functions and canonical pathways.

### Determination of miRNA and mRNAs Levels in Dorsal Hippocampus by RT-qPCR

#### Validation of miRNAs

RNA < 200 nt (100 ng) was polyadenylated and, at the same time, reverse transcribed using the miScript II RT kit (see footnote 1, QIAGEN, Hilden, Germany), according to the manufacturer’s instructions. This reaction was carried out for 1 h at 37°C and the enzyme was then inactivated by heating at 95°C for 5 min. The qPCR experiments were conducted as previously described on a Stratagene Mx3000p thermocycler (Stratagene, Agilent), using a denaturation step (95°C for 15 min), followed by 40 cycles of 94°C for 15 s, 55°C for 15 s and 70°C for 15 s (Castaneda et al., 2015). Each reaction was carried out in duplicate in a final volume of 25 μL and contained 12.5 μL miScript SYBR Green PCR, 2.5 μL of universal primer (see footnote 1, QIAGEN, Hilden, Germany), 2.5 μL of specific Primer Assay (QIAGEN, Hilden, Germany) and 200 pg of cDNA. A melting curve analysis was conducted afterward by heating the samples at 1°C per second from 70 to 95°C in order to detect PCR products. Designed primers were obtained from QIAGEN (Hilden, Germany). Primer sequences were 5′-UAUUGCACUUGUCCCGGCCUG-3′ for miR-92 and 5′-AGAGGCUGGCCGUGAUGAAUUC-3′ for miR-485, with an amplification efficiency of 89.5 and 86.8%, respectively. The relative abundance of these miRNAs was normalized to the levels of small nucleolar RNA SNO95, using standard primers (see footnote 1, QIAGEN, Hilden, Germany, cat # MS00033726, efficiency 97.3%), as we previously described (Castaneda et al., 2015). Relative miRNA levels were calculated based on the 2^−ΔΔCT^, normalized to that of the SNO95 gene cDNA, and then relativized to control unstressed animals (Castaneda et al., 2015). All RT and qPCR reactions included water and RNA without the RT reaction as controls.

#### Determination of mRNA Levels in Dorsal Hippocampus

RNAs > 200 nt were reverse transcribed into cDNA by using Superscript II (Invitrogen, Carlsbad, CA, United States), following the manufacturer’s instructions, but in the presence of RNAsin^®^ (40 U, Promega Corporation, Madison, United States) as RNAase inhibitor. The qPCR was conducted in duplicate with 10 μL Brilliant II Ultra-fast SYBR Green QPCR Master Mix (Agilent Technologies), a suitable dilution of cDNA and 0.12 μM of primers from Integrated DNA Technologies (Coralville, IA, United States) and designed with Primer-Blast, NCBI. To determine CREB mRNA levels, we used a forward primer 5′-GAGAACAGAGTGGCAGTGCT and a reverse primer 5′-GGTCCTTAAGTGCTTTTAGCTCC (XM_017596652.1) that generate an amplicon of 70 bp. Additionally, we determined β-actin housekeeping gene mRNA levels by using the following forward primer 5′-TTGTCCCTGTATGCCTCTGGTC-3′ and reverse primer 5′-ACCGCTCATTGCCGATAGTG-3′ (NM_031144.3), which generate an amplicon of 346 bp. The efficiency of each primer set was obtained with several inputs of cDNA, and specificity was validated through melting curve analyses. Relative mRNA levels were calculated based on the 2^−ΔΔCT^ and normalized to that of β-actin mRNA.

### Determination of CREB Protein Levels in Dorsal Hippocampus

Dorsal hippocampi were homogenized as we previously described (Pacheco et al., 2017), in the presence of 0.32 M sucrose, 1 mM calcium-magnesium chelators (EDTA/EGTA), protease inhibitor (Complete^TM^ EDTA-free, Sigma-Aldrich), and phosphatase inhibitor (PhosStop^TM^, Sigma Aldrich) buffered with 10 mM HEPES at pH 7.4. Samples were then centrifuged at low speed (430 ×*g* for 10 min at 4°C) to obtain a pellet (fraction enriched in nuclei) and a supernatant, which corresponds to a fraction of homogenate without nuclei. These fractions were mixed with loading buffer and then boiled during 10 min as described previously (Pacheco et al., 2017). Proteins (30 μg) were resolved on 10% SDS–PAGE and then blotted onto 0.2 μm PVDF membranes (2 h at 350 mA). Membranes were then incubated during 1 h at room temperature in 1% non-fat milk dissolved in PBS with 0.1% Tween-20 (PBS-T) and incubated overnight with a 1:500 dilution of CREB rabbit monoclonal antibody (catalog No 9197, Cell Signaling Technology). The blots were rinsed with PBS-T three times (each during 5 min) and then incubated at room temperature for 2 h with peroxidase-conjugated anti-rabbit secondary antibody (1:10,000, Thermo Scientific, Waltham, MA, United States). Membranes were then incubated with enhanced chemiluminescence Detection Kit for peroxidase (catalog No 20-500-120 Biological Industries, Israel Beit Haemek Ltd.) and signals were detected with a chemiluminescence imager (Syngene, United Kingdom). Blots were then treated with ReBlotPlus Mild Antibody Stripping Solution (Sigma-Aldrich, St. Louis, MO, United States) during 10 min and after were washed with PBS. Blots containing homogenate samples were blocked in 3% non-fat milk in PBS-T during 1 h and then incubated overnight with β-actin antibody in blocking solution (1:5000, Catalog No A5316, Sigma-Aldrich). Blots containing nuclear fraction were blocked in the presence of 1% BSA in PBS during 1 h and then incubated with antibody for Lamin B1 (LB1, monoclonal, 1:300, Catalog No sc-365962, Santa Cruz). Levels of β-actin and LB1 were used as loading controls for the homogenate fraction without nuclei and for the nuclear fraction, respectively. After capturing the images, band intensity was determined with the UN SCAN IT software (^4^RR ID: SCR_013725). Data represent the relative intensity ratio between CREB and respective loading controls (CREB/β-actin and CREB/LB1).

### Statistical Analysis

Statistical analyses were conducted using GraphPad Prism (GraphPad Software Inc., San Diego, CA, United States). Data represent the mean ± SEM. Considering that data did not show normality test distribution (D’Agostino-Pearson omnibus and Shapiro–Wilk test), the data were analyzed either by the non-parametric Kruskal–Wallis test, followed by Dunn’s *post hoc* test (for comparison between three groups) or the Mann–Whitney *U* test (to compare two experimental groups).

## Results

### Effectiveness of Repeated Restraint Stress Through Evaluation of Physiological Markers

**FIGURE 2:**
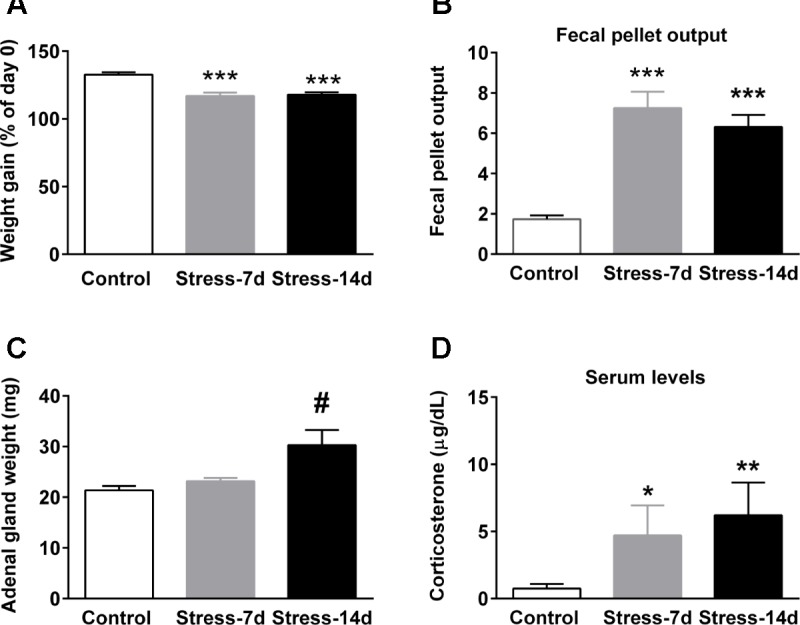
Effect of restraint stress on body weight gain, fecal pellet output, adrenal gland weight, and baseline corticosterone levels. **(A)** Variation of body weight gain was evaluated at the end point of treatment. All rats were weighed daily and one group was stressed during 14 consecutive days. The graph represents the change in body weight as a percentage of the initial weight. **(B)** Fecal output was determined every day in control (2.5 h) and stressed animals during the stress session. Data represent the mean value/day. **(C)** Adrenal gland weight of control and stressed animals. **(D)** Serum corticosterone levels in control and stressed animals. Blood was sampled between 9:30 and 11:00 a.m. All data represent mean ± SEM of Control, *n* = 16 and animals subjected to restraint stress during 7 (Stress-7d, *n* = 9) and 14 days (Stress-14d, *n* = 17). Dunn’s *post hoc* test: ^∗∗∗^*P* < 0.001, ^∗∗^*P* < 0.01, ^∗^*P* < 0.05, Mann–Whitney test two-tail, ^#^*P* < 0.05.

The effectiveness of repeated stress was confirmed by using several physiological parameters, and the data represent pooled animals that were used for miRNA array, RT-qPCR analyses and western-blot analysis. **Figure 2A** shows body weight gain at the experimental end-point. Kruskal–Wallis analysis revealed differences between groups (*P* < 0.0001) and Dunn’s *posthoc* test revealed that controls increased their initial weight by 35%; in contrast to stressed animals during 7 (Stress-7d) and 14 days (Stress-14d), that gained approximately 17% (*P* < 0.001) and 18% (*P* < 0.001), respectively. We also determined fecal output every day during the stress procedure as a readout of stress effectiveness (Nakade et al., 2007), and estimated the mean *per* stress session. We found significant differences between groups (Kruskal–Wallis, *P* = 0.0002) and Dunn’s *post hoc* test indicated a rise in the number of fecal pellets voided in both stress groups (both *P* < 0.001), compared to unstressed animals (**Figure 2B**). Furthermore, we determined that the weight of adrenal glands did not show differences among groups (Kruskal–Wallis, *P* > 0.1); nonetheless, we did observe an increase in Stress-14d when compared to controls (Mann–Whitney test, **Figure 2C**, ^#^*P* < 0.05). Blood samples for CORT determination were obtained 24 h after the last stress session and in basal conditions (blood sampled between 9:30 and 11:00 a.m.). Kruskal–Wallis analysis revealed significant differences between groups (*P* = 0.005) and Dunn’s *post hoc* test showed that both the Stress-7d and -14d groups had high baseline CORT levels compared to the control group (*P* < 0.05 and *P* < 0.01, respectively; **Figure 2D**). Altogether, these data indicate that restraint stress triggers the activation of the HPA axis and that the rats did not adapt when exposed daily to the homotypic stressor.

### The miRNAs Are Up- and Down-Regulated in Dorsal Hippocampi of Stressed Animals

**FIGURE 3:**
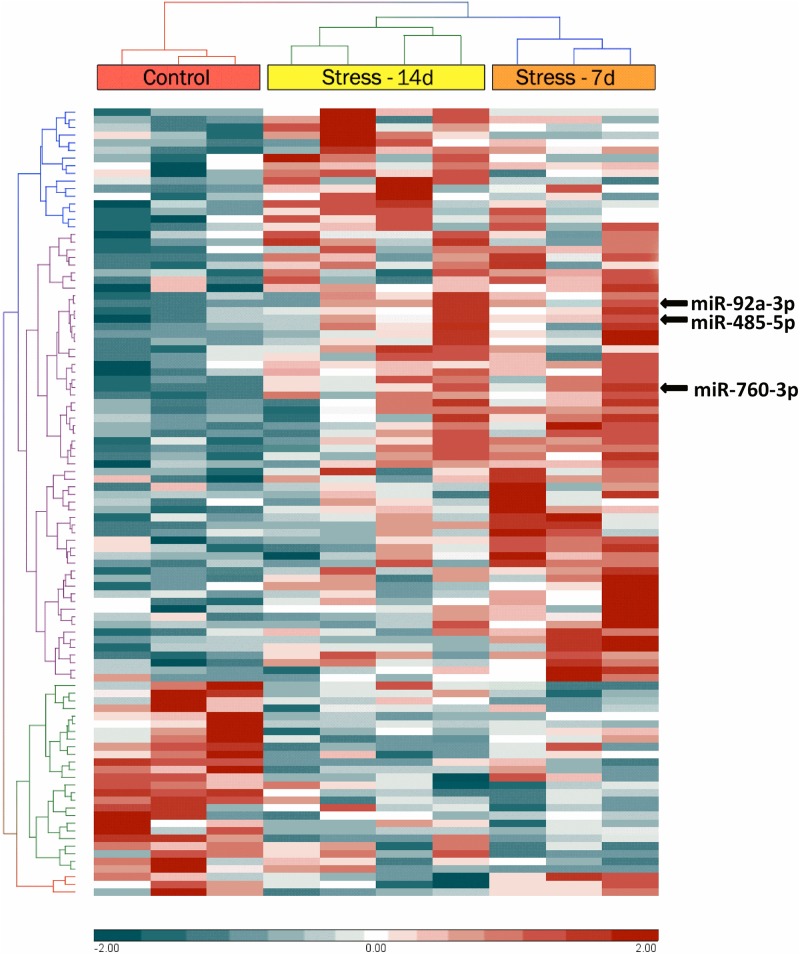
Hierarchical cluster analysis of microRNAs (miRNAs) with significantly altered expression in rat dorsal hippocampus, representing the time evolution of stress treatment. Hierarchical clustering was performed on all samples of miRNAs differentially expressed between control dorsal hippocampi samples with fold changes greater than 2 (red) or less than –2 (blue). The top bar indicates experimental group [red: control (*n* = 3); orange: stressed animals during 7 days (Stress-7d, *n* = 3); yellow: stressed animals during14 days (Stress-14d, *n* = 4). Each row identifies one miRNA (not listed).

Dorsal hippocampi obtained 24 h after the last stress session were used for microarray analyses to detect the miRNA expression profile. A two-way hierarchical clustering analysis of controls and stressed animals showed significant variations of 103 miRNAs (*P*-value < 0.05) (**Figure 3**). This analysis segregated animals in two main clusters: one branch included control animals and the other one, stressed animals (during 7 and 14 days). We noticed that the miRNA profile of stressed animals during 7 days was very different from that of the controls, but more similar to the profile corresponding to animals stressed during 14 days (**Figure 3**). We used two bioinformatic tools in order to refine the search for significant miRNAs. Limma analysis revealed that after 7 days of stress, only 92 miRNAs changed their levels; on the other hand, Partek analysis detected changes in 78 miRNAs. To make the analysis more robust, we only selected those miRNAs that merged common to both analyses, thus detecting 71 miRNAs (**Supplementary Table S1**). Interestingly, our analysis revealed variations in the levels of 51 mature miRNAs and 20 precursor miRNAs (stem-loop). Most stem-loops (15 out of 20) increased their levels, and only a few were accompanied by changes in the levels of its respective mature form (miR-3594-3p; miR-598-5p). Similarly, many mature miRNAs increased their levels (54 out of 71) after 7 days of stress. Limma analysis also showed that after 14 days of stress, 64 precursors and miRNAs vary significantly, 55 of which were also found under Partek analysis (**Supplementary Table S2**). We found that 17 of these correspond to stem-loop (8 increased and 9 were reduced) and that the remnant are mature forms (12 increased and 26 were decreased). We also detected that variations in stem-loop miRNAs levels were correlated with significant variations in their mature forms (miR-24-1-5p, miR-296-5p, and miR-328a-3p). Interestingly, we detected variations in the levels of several miRNAs that belong to the miR-370-410 cluster at both stress periods.

**FIGURE 4:**
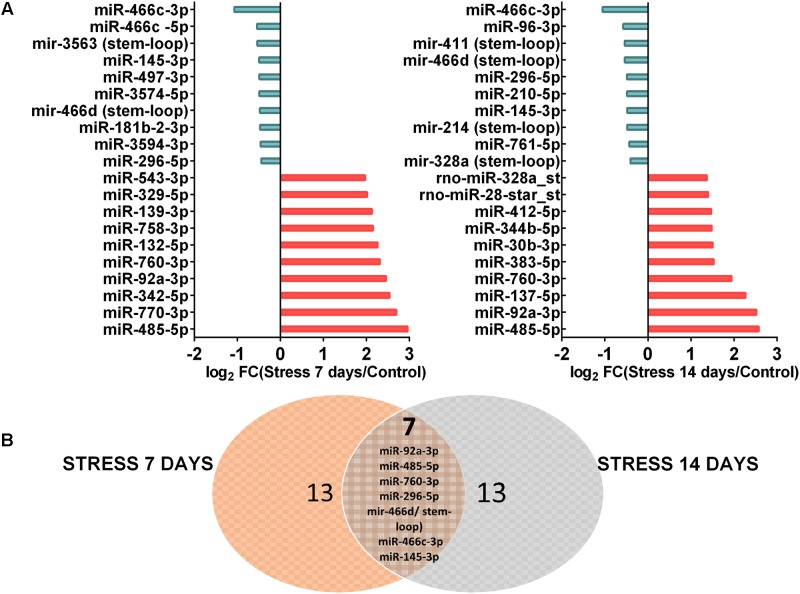
Top 10 miRNAs most differentially expressed in dorsal hippocampi after 7 and 14 days of stress. **(A)** Distributions of Top 10 up-regulated miRNAs and down-regulated miRNAs after 7 and 14 days of stress in comparison to control animals, expressed as log2 FC. **(B)** The Venn diagram showing the overlapping between dorsal hippocampus of stressed animals during 7 days (left, orange circle) and 14 days (right, light blue circle).

These data indicate that a lower number of miRNAs changed their levels after 14 days of stress, in comparison to 7 days of stress. Thus, it is possible that stress triggers changes in the expression, processing and/or turnover of particular miRNAs. We segregated 10 miRNAs according to their maximal variation respective to control. These top-10 miRNAs are shown in **Figure 4A**, which indicates that the magnitude of reduction in their levels (log_2_ FC) was lower than that of increase. It is noteworthy that at both stress periods, the miRNAs that reduced their levels were different. Furthermore, Venn diagram revealed seven common miRNAs that changed their levels at both stress periods (**Figure 4B**). Of those, three miRNAs (miR-296-5p, miR-466c-3p, miR-145-3p) and one precursor (miR-466d/stem-loop) decreased their levels; in contrast, three miRNAs (miR-760, miR-92a, and miR-485) increased their levels in dorsal hippocampus after both 7 and 14 days of stress (**Figures 3**, **4**). Thus, it is plausible that the chronicity of the stressor establishes waves of miRNA expression, and that some changes persist after 7 days of stress.

### Validation of Selected miRNAs by qPCR

**FIGURE 5:**
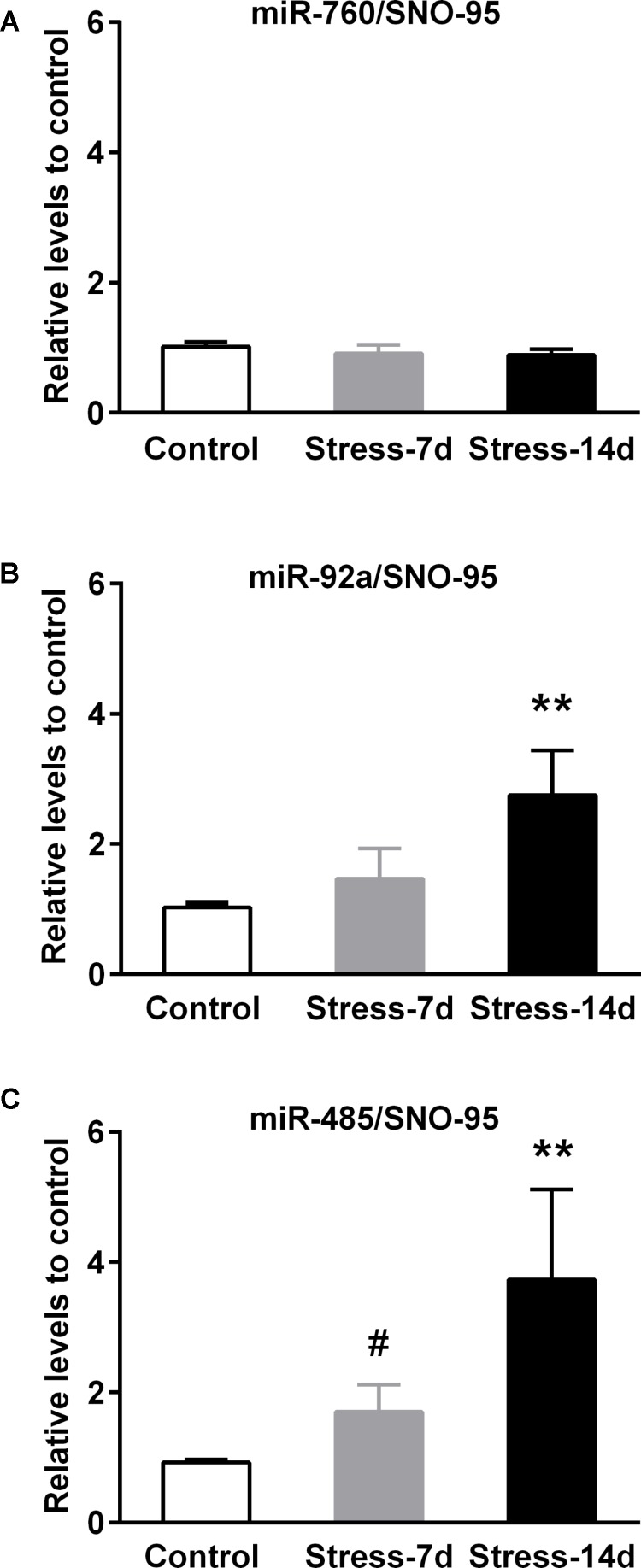
Validation of stress-induced miRNAs expression in dorsal hippocampus. The miR-760, miR-92a, and miR-485 levels were determined by quantitative real-time PCR (qPCR) after 7 and 14 days of restraint stress. **(A)** Levels of miR-760 in dorsal hippocampi of Control (*n* = 7), Stress-7d (*n* = 5) and Stress-14d (*n* = 7) animals. **(B)** Levels of miR-92a in Control (*n* = 7), Stress-7d (*n* = 4) and Stress-14d (*n* = 8) group. **(C)** Levels of miR-485 in Control (*n* = 4), Stress-7d (*n* = 5) and Stress-14d (*n* = 5). Data were analyzed by Kruskal–Wallis test followed by Dunn’s *post hoc* test, ^∗∗^*P* < 0.01. Mann–Whitney test two-tail, ^#^*P* < 0.05.

We next validated the changes in miR-760-3p, miR-92a-3p, and miR-485-5p by qPCR on independent biological samples obtained after 7 and 14 days of stress, using RNA expression of SNO95 gene as normalizer, as we previously validated (Castaneda et al., 2015). We detected that miR-760 levels were insensitive to both stress periods (**Figure 5A**). In contrast, the analysis of miR-92a levels showed differences between groups (Kruskal–Wallis *P* < 0.005) and we found that the levels of miR-92a in stressed animals during 14 days increased twofold in comparison to controls (Dunn’s post-test *P* < 0.01, **Figure 5B**). Similarly, the expression of miR-485 was different among experimental groups (Kruskal–Wallis, *P* < 0.01), and we detected a rise of almost threefold in animals stressed during 14 days in comparison to controls (Dunn’s post-test *P* < 0.01, **Figure 5C**). However, the difference between controls and animals stressed during 7 days was only detected by Mann–Whitney analysis, and indicated a 1.5 fold increase in miR-485 levels (one-tailed test *P* < 0.05).

**FIGURE 6:**
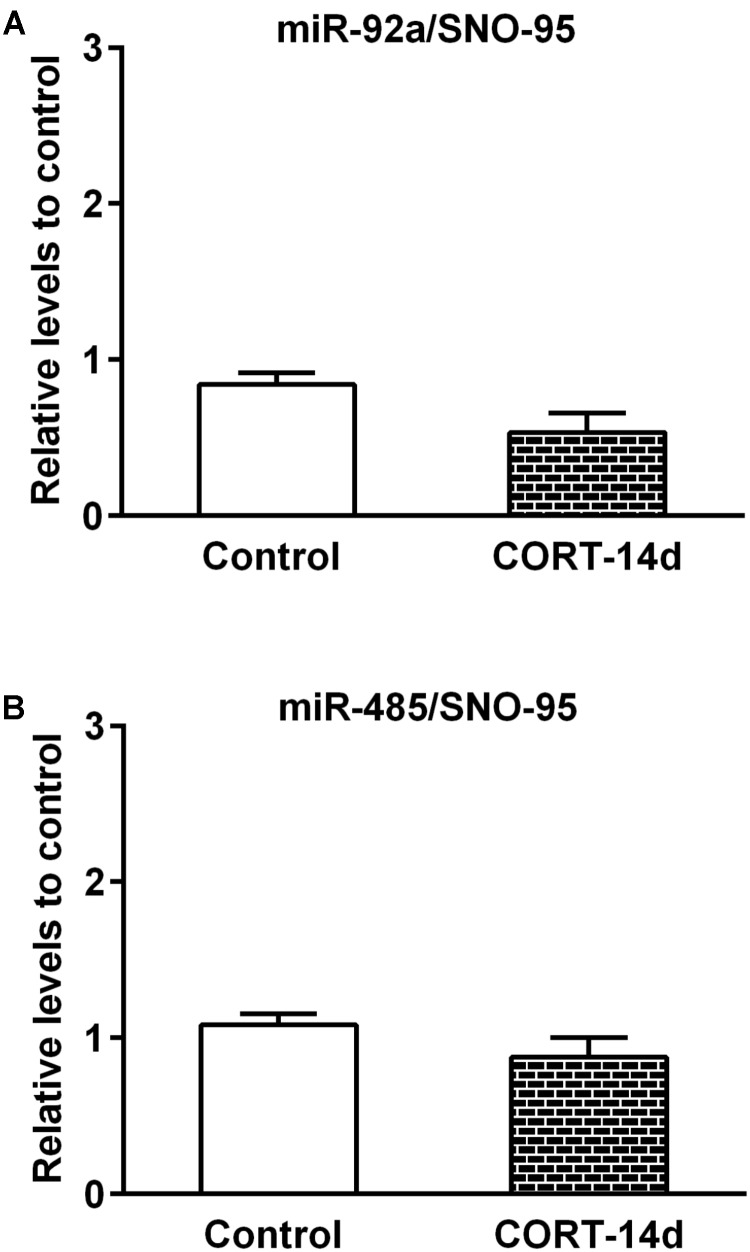
Effect of corticosterone administration during 14 days on miR-92a and miR-485 expression in dorsal hippocampus. The miRNAs levels were determined by quantitative real-time PCR (qPCR) after daily 30 mg/kg s.c. administration of corticosterone (CORT) or vehicle (Propylene glycol). **(A)** Levels of miR-92a in Control (*n* = 5) and CORT-treated (*n* = 5) animals. **(B)** Levels of miR-485 in control (*n* = 6) and CORT-treated (*n* = 5) animals.

In order to understand whether some of the stress mediators have a role in the expression of miR-92a and miR-485, we next examined the effect of chronic exogenous CORT administration (14 days) at a dose that we previously reported to induce an increased immobility time in the forced swimming test; i.e., similarly to our chronic stress model (Ulloa et al., 2010). Compared with vehicle control rats, CORT administration induced a reduction in both weight gain and adrenal gland weight, which is indicative of CORT effects (see **Supplementary Figure S1**). As shown in **Figure 6**, neither miR-92a nor miR-485 vary their expression under this CORT administration regimen. In whole, these results indicate that 14 days of stress triggers changes in the expression of miR-92a and miR-485 in dorsal hippocampi; variations that are not mimicked by CORT administration.

### *In silico* Studies to Search Targets and Putative Pathways Regulated by miR-92a-3p and miR-485-5p

**FIGURE 7:**
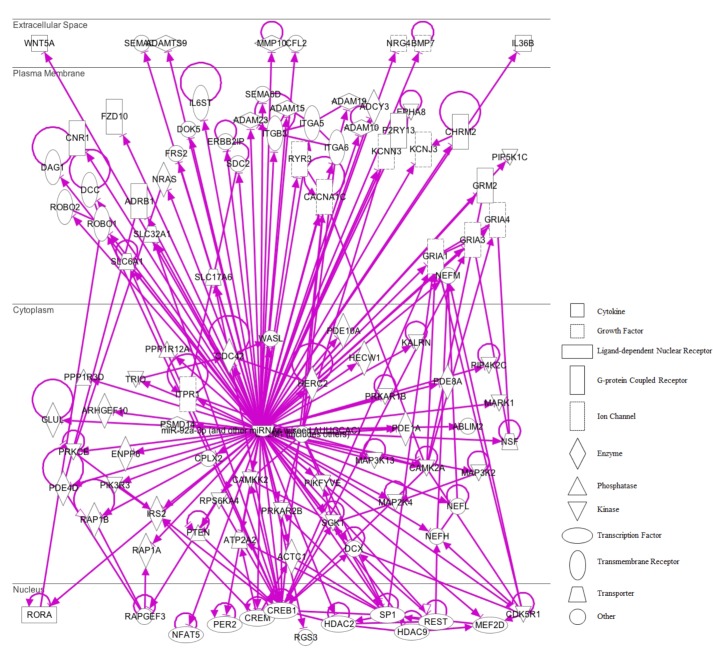
IPA pathway representation of miR-92a targets. 103 predicted and probed targets mRNAs are represented. Gene products are positioned according to subcellular localization. Only direct connections (i.e., direct physical contact between two molecules) among the individual gene products are shown; arrow-lines indicate protein-protein binding interactions.

**FIGURE 8:**
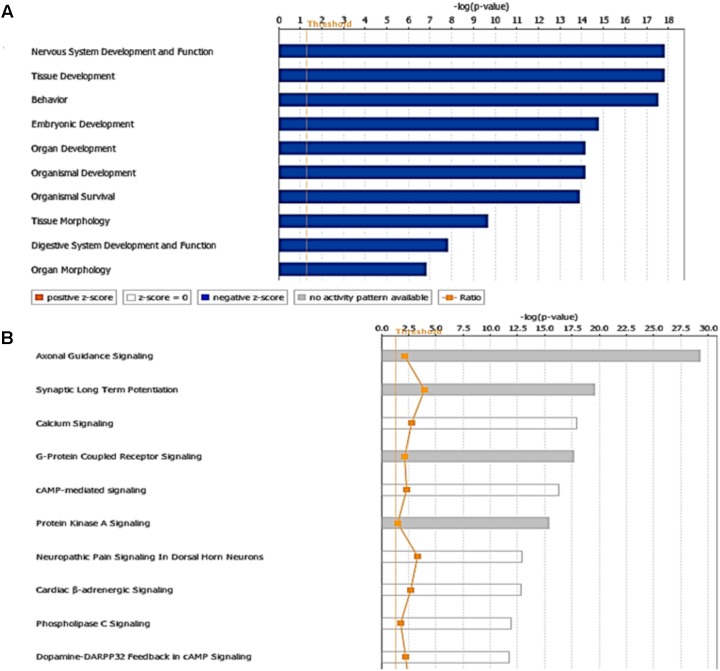
Targets and pathways influenced by miR-92a-3p. Targets were evaluated using Target scan and the pathways associated with genes that are predicted to be targets of miR92a are shown (*P* < 0.05, Fisher’s Exact test). **(A)** Top-10 biological functions identified by Ingenuity pathway analysis showing physiological and pathological functions that may be influenced and **(B)** Canonical pathways associated to miR92a (*P* < 0.05, Fisher’s Exact test). The ratio was determined as the number of genes in a given pathway divided by the number of genes that make up the pathway. The *P*-value for a given process annotation is calculated by considering the number of genes that participate in a process and the total number of genes that are known to be associated with that process in the selected reference set. Significance of upregulated or downregulated pathways was determined using Fisher’s exact test and is presented as the negative logarithm of the *P*-value [–log (*P*-value)]. A multiple corrections test is not available for IPA; therefore, all values are reported as unadjusted *P*-values. The predicted activation state (upregulated or downregulated) of significantly expressed pathways was determined by a *z*-score algorithm that compared the gene expression data set with the expected canonical pathway patterns (http://ingenuity.force.com/ipa). Pathways with positive (orange) and negative (blue) *z*-scores indicate that the pathways are activated and inhibited, respectively. Gray indicates that there is no report. Ratio is calculated as the number of genes that overlap with the corresponding pathway.

The miRNA Target Filter from IPA was used to gain insight about the gene products interaction network, their participation in canonical pathways, biologic functions, and cellular processes modulated by miRNA actions. From a total of 103 targets, we found 12 mRNAs with a moderate prediction, 86 with high prediction, and 5 experimental validated targets of miR-92a-3p (**Supplementary Table S3**). These targets were segregated according to their corresponding cellular compartments; 7 gene products were present at the extracellular space and correspond to peptides that are involved in cell polarity, and others correspond to secreted enzymes involved in matrix remodeling. Another main group of targets corresponded to proteins located at the plasma membrane, such as G-coupled receptors, subunits of channel receptors, scaffold proteins, and enzymes, among others (**Figure 7**). Furthermore, another group included targets located in the cytosol compartment, including phosphatases and other enzymes (**Figure 7**). Moreover, miR-92a had 13 predictive targets that correspond to transcription factors (**Figure 7**). *In silico* analysis revealed that the top-10 biological functions of miR-92a were related to: Nervous System Development and Function, Tissue Development, Behavior, Embryonic Development, Organ Development, Organismal Development, Organismal Survival, Tissue Morphology, Digestive System Development and Function, and Organ Morphology (**Figure 8A**). Target analysis revealed a large number of genes associated with canonical pathways related to Axonal Guidance Signaling, Synaptic Long-Term Potentiation, Calcium signaling, G-protein Coupled Receptor Signaling, cAMP-mediated signaling, PKA signaling, neuropathic pain signaling, cardiac β-adrenergic signaling, Phospholipase C signaling, and Dopamine-DARPP32 feedback in cAMP signaling (**Figure 8B**).

### *In silico* Studies to Search Targets and Putative Pathways Regulated by miR-485-5p

**FIGURE 9:**
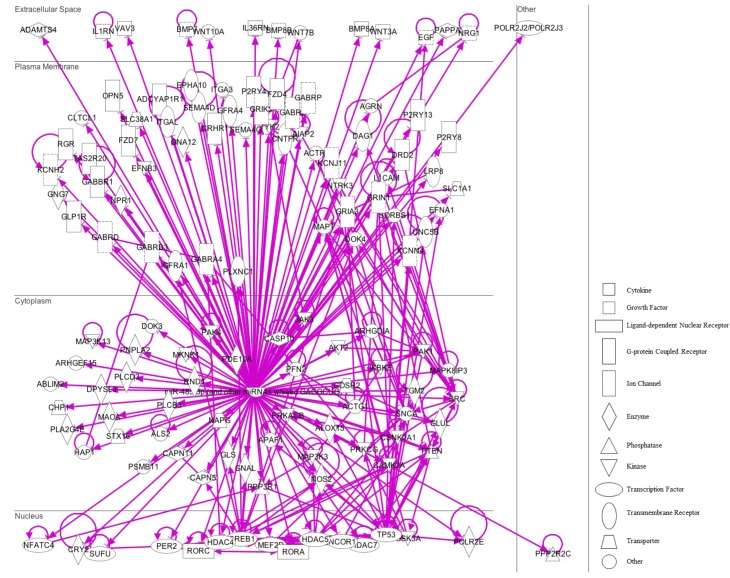
IPA pathway representation of miR-485 targets. 131 predicted targets mRNAs are represented. Gene products are positioned according to subcellular localization. Only direct connections (i.e., direct physical contact between two molecules) among the individual gene products are shown; arrow-lines indicate protein-protein binding interactions.

**FIGURE 10:**
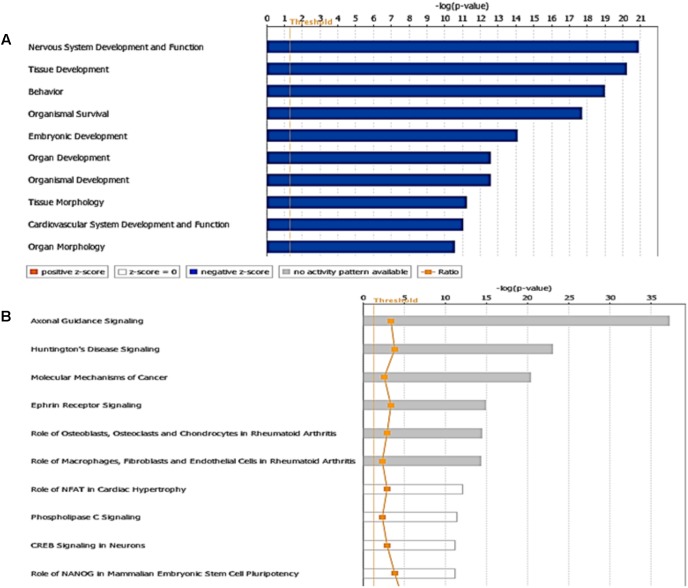
Targets and pathways influenced by miR-485-5p. **(A)** Top-10 biological functions identified by Ingenuity pathway analysis and **(B)** Canonical pathways associated to miR-485a (*P* < 0.05, Fisher’s Exact test). The ratio is calculated as the number of genes in a given pathway divided by the number of genes that make up the pathway. The *P*-value for a given process annotation is calculated by considering the number of focus genes that participate in that process and the total number of genes that are known to be associated with that process in the selected reference set. The predicted activation state (upregulated or downregulated) of significantly expressed pathways was determined by a *z*-score algorithm that compared the gene expression data set with the expected canonical pathway patterns (http://ingenuity.force.com/ipa). Pathways with positive and negative *z*-scores indicate that the pathways are activated and inhibited, respectively. Orange color indicates activation of the pathway, blue indicates suppression of the pathway and gray indicates a mixed response. Ratio is calculated as the number of genes that overlap with the corresponding pathway.

On the other hand, in the case of miR-485-5p, we found 70 target mRNAs with moderate prediction and 61 with high prediction, from a total of 131 targets (**Supplementary Table S4**). Fourteen gene products were present at the extracellular space and correspond to morphogen peptides, Wnt signals, growth factors, and extracellular proteases, among others (**Figure 9**). Furthermore, a large number of gene targeted products (50 mRNAs) are G-coupled receptors, subunits of channel receptors, transporters, adhesion molecules, enzymes, and scaffold proteins (**Figure 9**). The other group corresponded to 45 targets located in the cytosol and includes enzymes such as kinases and phosphatases, among others. Interestingly, there are many transcription factors, along with HDAC and ligand-dependent nuclear receptors, which are predicted to be miR-485 targets. Analysis of the top-10 canonical pathways influenced by miR-485 detected 9 of the same 10 pathways predicted for miR-92, with the exception of Digestive System Development and Function, but including Cardiovascular System Development and Function (**Figure 10A**). Target analysis for miR-485 revealed a large number of genes associated with canonical pathways related to Axonal Guidance Signaling (i.e., similarly to miR-92a), Huntington’s Disease Signaling, Molecular Mechanism of Cancer, Ephrin Receptor signaling, Role of Osteoblasts, Osteoclasts and Chondrocytes in Rheumatoid Arthritis; Role of Macrophages, Fibroblasts and Endothelial Cells in Rheumatoid Arthritis, Role of NFATs in Cardiac Hypertrophy, PLC Signaling, CREB Signaling in Neurons and Role of homeobox protein NANOG in Mammalian Embryonic Stem Cell Pluripotency (**Figure 10B**).

### Relationship Between miR-92a-3p and miR-485-5p Target Genes

**FIGURE 11:**
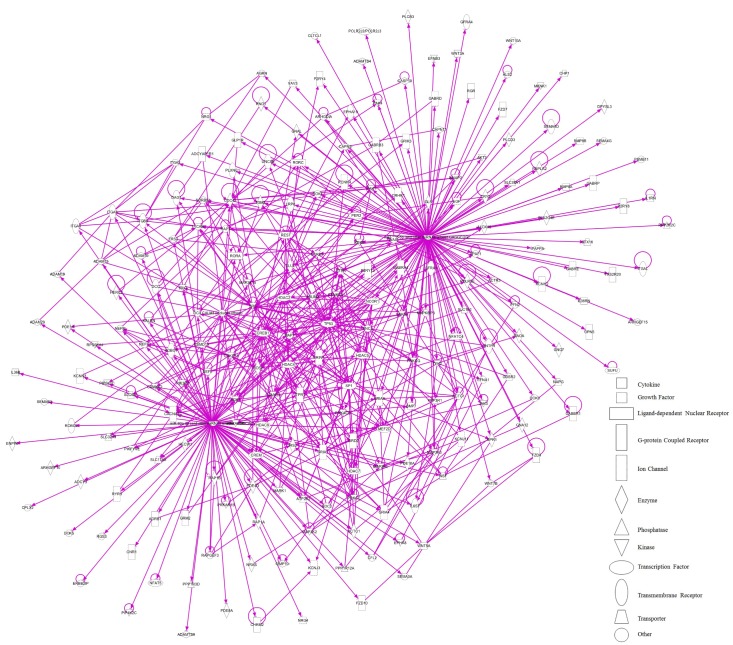
Proposed network regulated by variation of miR-92a and miR-485 induced by chronic stress in dorsal hippocampus. The molecular crosstalk (represented as solid lines for direct relationship) is shown as part of the stress-induced up-regulation of miR-92a and miR-485. Protein as part of a node can be observed such as HDAC 2, 4, and 5; CREB1; the tumor protein 53 (TP53); the nuclear receptor co-repressor 1 (NCOR1), synuclein alpha (SNCA), the microtubule associated protein Tau (MAPT) and the NR1 subunit of NMDA receptor (GRIN1).

**FIGURE 12:**
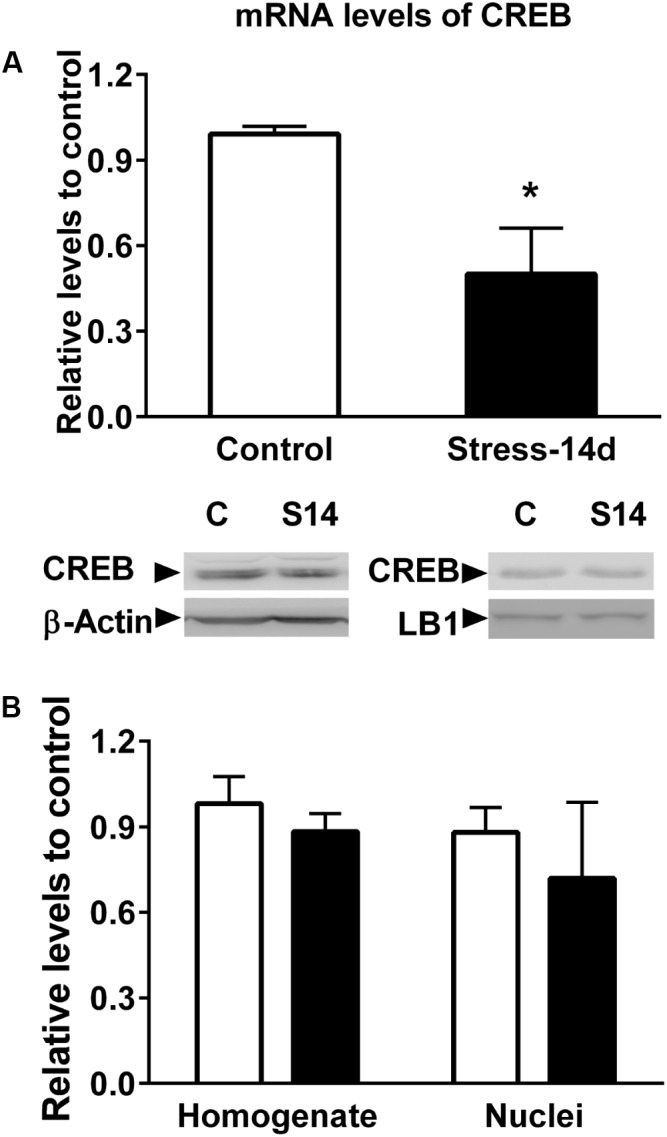
Evaluation of CREB mRNA and protein levels in dorsal hippocampus. **(A)** Comparison of CREB mRNA levels in control and chronically stressed animals. RT-qPCR experiments were conducted to determine CREB mRNA levels and data were analyzed by 2^−ΔΔCt^ using β-actin as normalizer. Bar graph represents mean ± SEM of Control (*n* = 6) and stressed animals (*n* = 6). Differences between both groups were evaluated with two-tailed Mann–Whitney test. ^∗^*P* < 0.05. **(B)** Levels of CREB protein levels in homogenate and nuclear fraction obtained from dorsal hippocampus. β-actin and Lamin B1(LB1) were used as loading controls for homogenate and nuclear fractions, respectively. Graphs represent mean ± SEM of each group. Control (*n* = 6) and stressed animals (*n* = 4).

Further analysis centered on regulatory relationships between miR-92a and miR-485 target genes displayed some nodes with proteins that have a pivotal role in brain plasticity (**Figure 11**). These include HDACs (HDAC 2, 4, and 5); CREB1; the TP53; the NCOR1, SNCA, the MAPT and the NR1 subunit of the NMDA receptor (GRIN1). **Figure 11** indicates that these gene products are either connected directly or indirectly to each other. Considering the pivotal role of CREB in hippocampal function (Chen et al., 2001; Carlezon et al., 2005), we decided to determine variations in both its mRNA and protein levels in dorsal hippocampus after 14 days of stress. As shown in **Figure 12A**, CREB mRNAs levels decreased with stress (two-tailed Mann–Whitney analysis, *P* < 0.05); nonetheless this variation was not related to a similar variation in hippocampal protein extract (without nuclei) and nuclear fraction (**Figure 12B**).

## Discussion

Stress triggers changes in the activity of several neurocircuits in diverse brain areas, particularly in the hippocampus. Antecedents point out that the intensity, duration and chronicity of exposure to stressors determines how the hippocampus reacts to this challenge (Joels and Baram, 2009). We have previously reported that chronic restraint stress in rat induces anhedonia, depressive- (Bravo et al., 2009; Castaneda et al., 2015; Pacheco et al., 2017) and anxiety-like behaviors (Bravo et al., 2009; Ulloa et al., 2010). Moreover, a recent systematic review and meta-analysis conducted in rodents indicated that different models of chronic stress impair the consolidation of learned memories (Moreira et al., 2016).

One of the most important findings of the present study was that the extent of stress exposure (7 vs. 14 days) triggers a differential response in miRNA levels at dorsal hippocampus and remarkably, we found that some of these miRNAs belong to the miR-379-410 cluster. Moreover, we confirmed by qPCR that miR-92a and miR-485 are induced after 14 days of stress in dorsal hippocampus. Furthermore, chronic administration of CORT during 14 days, used to emulate variations in hormone levels that occur during stress exposure, did not mimic the effect of restraint stress; suggesting that the expression of these miRNAs are independent of the effect of this stress hormone. These data indicate that stress triggers changes in the levels of master regulators of mRNAs translation, probably by producing changes in the efficacy of some important transduction signaling pathways in dorsal hippocampus, and hence, determining several neuroplastic changes and functioning of this structure.

### The Extent of Repeated Stress Triggers Changes in miRNAs in Dorsal Hippocampus

Evidences recently reviewed indicate that environmental stress exposure may trigger changes in miRNA levels (Hollins and Cairns, 2016). Studies in the prefrontal cortex of adult mice indicated a prominent increase in the levels of different miRNAs after acute stress (2 h) while only minor changes were observed after subchronic restraint (5 days) (Rinaldi et al., 2010). Moreover, these changes were region specific, i.e., with no differences in miRNAs expression in the hippocampus (Rinaldi et al., 2010). In adult male rats, acute immobilization stress triggers an increase in particular miRNA levels, which are reduced after chronic stress in hippocampus and amygdala (Meerson et al., 2010).

The aforementioned antecedents suggest that acute, subchronic, and chronic stress can differentially modify miRNA levels according to specific patterns. With the idea of contributing to this important issue, we chose 7 and 14 days of stress exposure, a particular timing in which we previously observed anhedonic behavior (Castaneda et al., 2015). We observed HPA axis activation (reduction in weight gain, and increase in both fecal output and baseline CORT levels) in both stressed groups, with no sign of adaptation to the homotypic stressor exposure. We used the Affymetrix platform for miRNA expression analysis and mainly detected a rise in miRNA levels and also in some miRNA precursors; variations that may be linked to a stress-induced gene expression profile. It is also important to note that the heatmap miRNAs analysis revealed that miRNA profiling of animals stressed during 7 days was different from that of controls; in contrast, the profile of rats stressed during 14 days was more similar to controls and animals stressed during 7 days. Thus, it is probable that during the first week of stress, more miRNA genes respond to this challenge, and that some compensatory mechanism is triggered to produce a new profile with a lower number of miRNAs that change after 14 days of stress. To sum up, miRNA profiling in dorsal hippocampus depends on the extent of stress exposure and future prospects will consider determining transcriptional, and post-transcriptional mechanisms associated to the stress-induced regulation of miRNA expression.

We were able to confirm by RT-qPCR only two of the three miRNAs that showed the highest increase after 14 days of stress according to chip analysis (miR-92a and miR-485). The discrepancy between the array and qPCR data may depend on a wide repertoire of factors, such as the inherent drawbacks of both techniques and the different normalization and dynamic range of each technique, among others (Morey et al., 2006; Koshiol et al., 2010).

Considering that the chronic stress paradigm is accompanied by a rise in CORT levels, we decided to examine the direct effect of daily administration (14 days) of this hormone on miR-92a and miR-485 levels. Under chronic CORT administration, we have previously observed a depressive-like behavior (high time in immobility in the Forced-swim test) (Ulloa et al., 2010). Additionally, other studies using chronic administration of CORT indicated a reduction in sucrose preference, decreased reward behavior and impaired spatial working-memory (Sterner and Kalynchuk, 2010). In the present study, chronic CORT administration produced a reduction in adrenal gland weight and body weight gain, but did not vary miR-92a and miR-485 levels in dorsal hippocampus. Considering that we do not know how chronic CORT exposure may influence miRNA profiles, we can only conclude that the levels of miR-92a and miR-485 levels are independent of this hormone. Taken altogether, it is plausible that even though the expression of miR-92a and miR-485 is stress-dependent, but CORT independent, they may regulate either the basal or stress-induced variation in the expression of several genes.

### The Predicted Targets for miR-92a and miR-485 Are mRNAs Involved in Neuronal Function

The miR-92a-3p is preferentially expressed in glutamatergic neurons (He et al., 2012), and belongs to a family of highly conserved miRNAs with an identical seed region found in diverse paralog clusters: miR-17-92, miR-106a-363, and miR-106b-25 (Tan et al., 2014). A rise in several members of the miR-17-92a cluster (miR-92a, miR-20a) has been detected in brain samples of patients with Huntington’s disease (Marti et al., 2010). Interestingly, after stroke, intravenous administration of exosomes containing members of the miR-17-92 cluster promotes neurite branching, as well as a rise in dendritic spine density in the infarcted brain area of rats (Xin et al., 2017); nonetheless, it is not clear whether a particular miRNA of this cluster is responsible for this effect. On the other hand, deletion of the miR-17-92 cluster in neural progenitors reduces neurogenesis in DG of hippocampus; which was linked to a rise in SGK1 (Li et al., 2016). The GO analysis of targeted mRNAs showed that many of these mRNAs are related to cell proliferation and differentiation (Jin et al., 2016). Additionally, deletion of this cluster promoted depressive-like behaviors, suggesting that members of the cluster may produce antidepressant-like effects (Jin et al., 2016); nonetheless, it is difficult to elucidate which member of this cluster is responsible for the observed effect.

We observed that miR-92a expression is enhanced after 14 days of stress in dorsal hippocampus; a variation that was not accompanied by similar changes in other members of the miR-17-92a cluster (see **Supplementary Tables S1** and **S2**). Nonetheless, we cannot discard the possibility that the different miRNAs from the cluster may have different half-lives (Ruegger and Grosshans, 2012). In cultured rat hippocampal neurons, pharmacological blockage of neuronal excitability triggers a decrease in miR-92a levels, favoring the rise in levels of its target, the subunit GluA1 of AMPA receptor (Letellier et al., 2014). We have recently reported that chronic stress does not change GluA1 levels in dorsal hippocampus (Pacheco et al., 2017), probably ruling out the contribution of miR-92a in the regulation of GluA1 levels. Additionally, contextual fear conditioning in mice triggers a transient rise in miR-92 levels in the hippocampus and reduces various mRNA targets, such as the neuronal K^+^/Cl^−^ co-transporter 2 (KCC2); CPEB3, a translational regulator in neurons; and the transcription factor MEF2D (Vetere et al., 2014). Additionally, reduction of endogenous miR-92 levels in CA1 neurons prevents variation in these proteins, restricts contextual fear conditioning, and prevents the rise in spine density triggered by memory formation (Vetere et al., 2014). Under this view, it seems that enhanced miR-92a expression may act as compensatory mechanism by limiting the reduction of spine density induced by stress in CA1 (Castaneda et al., 2015; Garcia-Rojo et al., 2017). Additionally, the IPA pathway analysis that we conducted identified canonical pathways associated with miR-92a, such as synaptic LTP, which is a process affected in chronically stressed animals (Pavlides et al., 2002; Alfarez et al., 2003).

We have also shown that some miRNAs residing in the miR379-410 cluster and other stem-loop precursors were mainly up-regulated under stress, suggesting that the expression of this cluster is stress-sensitive. Notably, we detected that stress triggers a rise in the levels of other members of the cluster at 7 days (miR-485, miR-770, miR-758, miR-543, miR-433, miR-154, miR-377, miR-666) and 14 days (miR-485, miR-412, miR-410, miR-127, miR-323, miR-44, miR-411) of stress, and that a few of them were expressed at both stress periods (such as stem-loops miR-300 and miR329). Interestingly, the miR-379-410 cluster, exclusively expressed from the maternal allele, is implicated in diverse neurodevelopmental processes and is a central regulator of neuronal function (Winter, 2015). The expression of this cluster is regulated by neuronal depolarization in a MEF2- and BDNF-dependent manner (Fiore et al., 2009) and at least three of its members (miR-134, miR-329, and miR-381) are crucial for dendritic spine formation induced by neuronal activity (Fiore et al., 2009). Interestingly, we have found that miR-485 levels increase during both of the stress periods evaluated in this study. On the other hand, the increase in neuronal activity of cultured neurons promotes a destabilization of mRNAs that have a common motif in the 3′UTR complementary to the seed domain of several miRNAs, including miR-485 (Cohen et al., 2014). Furthermore, miR-485 decreases TAU mRNA levels in hippocampal neurons, blocks nerve growth factor-induced neurite outgrowth in PC12 cells and reduces the levels of SV2A, a glycoprotein present in synaptic vesicles; suggesting that this miRNA may diminish neurotransmitter release (Cohen et al., 2011). Moreover, overexpression of miR-485 in cultured hippocampal neurons reduces the density of dendritic spines, along with the clustering of PSD-95 and the exposure of AMPA receptor containing GLUA2 subunits at the synaptic plasma membrane (Cohen et al., 2011); however, the mechanisms by which miR-485 produces this effect is unknown. We have recently reported that chronic stress does not produce changes in GLUA2 levels in homogenates obtained from dorsal hippocampus (Pacheco et al., 2017), suggesting that under stress conditions, miR-485 probably targets other mRNAs.

In order to gain insight about probable mRNA targets, we used gene networks based on the IPA tool and found that miR-485 is related to Huntington’s disease signaling. Recent evidence suggests a strong relationship between MDD and neurodegenerative diseases, including Huntington’s disease, as well as natural processes of aging [reviewed in Reus et al. (2016)]. On the other hand, our *in silico* study identified the top-10 biological functions influenced by miR-92a, nine of which were shared with miR-485 (Nervous System Development and Function, Tissue Development, Behavior, Embryonic Development, Organ Development, Organismal Development, Organismal Survival, Tissue Morphology, Digestive System Development and Function, and Organ Morphology). The only biological function distinctive of miR-92a was that related to Digestive System Development and Function. This global analysis points out that chronic stress may modify processes related to brain wiring that are fully active during development, but that it probably plays a key role in the adult brain. Interestingly, both miR-92a and miR-485 influence similar canonical pathways, such as axonal guidance signaling, which is relevant during development of the nervous system. This pathway involves the presentation of guidance signals, and both the reception and processing of these signals. The main extracellular signals that influence axonal growth cone dynamics include guidance signal families: NETRINS, SLITS, SEMAPHORINS (SEMA) and EPHRINS (EPH); and their corresponding neuronal receptors; i.e., DCC/UNC5, ROBO, NEUROPILIN/PLEXIN, and EPHS (Dickson, 2002). Some predicted targets of miR-92a actions include ROBO (1 and 2), SEMA3A and SEMA6D, DCC, and EPHA8. Although there is not much information about the role of axonal guidance signaling in MDD or stress-related disorders, some evidence indicates that alterations in these proteins are related to neurological disorders (Dickson, 2002). It is also important to highlight that different guidance molecules seem to have key roles in several aspects of synapse formation (Shen and Cowan, 2010) and dendritic spine stability (Koleske, 2013), a critical phenomenon in several animal models of depression (Castaneda et al., 2015; Pacheco et al., 2017). Interestingly, augmented EPHA4-EPHEXIN1 signaling in the prefrontal cortex and hippocampus has been related to depressive-like behavior observed after repetitive social defeat stress (Zhang et al., 2017), unveiling that pathways that canonically play a pivotal role during development may also be relevant in mature brain, perhaps favoring the development of some mood disorders.

### The Combined Rise in miR-92a and miR-485 Levels May Influence Gene Expression, Leading to Altered Neural Plasticity

Our *in silico* study also suggests that the combined effect of miR-92a and miR-485 on their targets may produce alterations in proteins related to neuronal functioning and we will now discuss three aspects especially related to the effect of stress on neuroplasticity. The first one is related to the possible influence of miRNAs on the levels of transcriptional factors. One of them is TB53, which is induced under chronic stress in the hippocampus (Sanchez-Hidalgo et al., 2016) and positively controls neuronal apoptosis (Jordan et al., 1997). On the other hand, the transcriptional factor CREB1—also included in this analysis—has a crucial role in the regulation of gene expression during the development of the nervous system and its expression is reduced in stressed animals (Marsden, 2013). In contrast, reports have shown that hippocampal CREB overexpression exerts antidepressant-like effects (Chen et al., 2001). In the present study, we detected a reduction in CREB mRNA levels in dorsal hippocampus that may be indicative of miRNAs action; nonetheless, we did not observe variations in CREB protein levels. However, there are many possibilities that can explain this discrepancy. For instance, we do not know whether chronic stress triggers changes in the half-life of the protein. On the other hand, since we only evaluated changes in dorsal hippocampus, we do not know whether variations in the levels of CREB protein could be related to a particular neuronal hippocampal stratum. Another possibility is that CREB is not controlled by these miRNAs. Prediction analysis indicated that both miR-92a and miR-485 might also influence gene expression by directly or indirectly targeting HDACs (2, 4, and 5). Some studies have indicated that HDAC2 negatively regulates memory formation, synaptic plasticity (Guan et al., 2009) and density of dendritic spines in hippocampal neurons (MacQueen et al., 2003); i.e., changes also observed under chronic stress (Castaneda et al., 2015; Pacheco et al., 2017). With respect to HDAC4, although it has no catalytic activity, it is able to repress the expression of genes that encode constituents of synapses; thus affecting synaptic architecture and strength (Sando et al., 2012). Additionally, *in vivo* suppression of HDAC4 in hippocampus has been reported to eliminate stress-induced altered behavior (Sailaja et al., 2012), suggesting that this protein has an important role in stress-related behavior (White and Wood, 2014). Considering the global effect of these HDAC in neuron physiology, it is plausible that the rise in both miR-92a and miR-485 may limit the negative effect of HDACs in hippocampal functioning.

The second aspect that is important to discuss concerns the stress-induced dendritic arbor retraction described in dorsal hippocampal neurons (Pinto et al., 2015) that may be related to changes in microtubule-associated proteins. Our analysis also detected the microtubule-associated protein TAU as part of an interaction node generated by miR-92a and miR-485. TAU interacts with tubulin and promotes microtubule stabilization (Avila, 2006); however, TAU knockout-mice do not show stress-induced altered behaviors and atrophy of hippocampal dendrites (Lopes et al., 2016). Considering these evidences, it is plausible that the expression of miR-92a and miR-485 may modulate the negative effect induced by stress.

The third aspect important to highlight is the description of the constitutive subunit of NMDA receptor (GRIN1) as a node of miR-92a and miR-485 interaction. In agreement, we have reported that chronic stress triggers a rise in the levels of GRIN1 mRNA, along with a reduction in protein levels in whole extract of dorsal hippocampus (Pacheco et al., 2017). Although we do not know the physiological consequence of this reduction, some studies have indicated that this subunit is not a restrictive factor in the number of NMDA receptors that are delivered from the endoplasmic reticulum to the synapse (Stephenson et al., 2008). These evidences suggest that stress-induced variation in miR-92a and miR-485 miRNA levels may regulate expression of important genes related to hippocampal physiology.

## Conclusion

Our study provides evidence that chronic stress triggers changes in the expression of miRNAs in dorsal hippocampus, in a way that is sensitive to the chronicity of the stress exposure. Further studies must be carried out not only to define a timeline of the spectrum of responses associated to repeated stress exposures—in which a transition of miRNA expression profile is produced—but also to determine whether these changes also involve a transition from homeostatic adaptation to a stress-induced condition characterized by poor adaptation. Understanding this transition will be relevant to gain insight about how stress regulates brain plasticity and pathology, especially in brain structures related to memory consolidation and cognitive functions, such as the hippocampus.

The other important finding is that miR-379-410 cluster expression is sensitive to stress, and the mechanisms by which some cluster members change their levels remains to be elucidated. Moreover, we also found that the stress-induced rise in miR-92a and miR-485 levels do not depend on CORT. Furthermore, we found that the pathways that seem to be mainly influenced by miR-92a and miR-485 are related to axonal guidance signaling and cAMP signaling. Additionally, the combined effect of miR-92a and miR-485 may influence transcription factors, along with histone-modifying enzymes, among others. Nonetheless, future studies must be focused to precise the hippocampus subfields and neuronal fate where these changes in miRNAs are occurring and whether they are limited to somatic or dendritic compartments. Similarly, the physiological relevance of the variations in these particular miRNAs should be addressed through interventional approaches, which undoubtedly may unveil whether or not these miRNAs influence dorsal hippocampus functioning. Altogether, these future scenarios will make it feasible to decipher whether the rise in miR-92a and miR-485 in dorsal hippocampus corresponds to a compensatory or maladaptive stress response.

## Author Contributions

MML, MGP, and JLF designed the experiments. MML, MGP, AP, GGR, and EV performed the experiments. MML, XX, TM, and FA analyzed and interpreted the data. FA, MTB, and JLF wrote the paper. RG, JAC, PR, and EA revised critically the manuscript. All authors edited drafts and approved the final version.

## Conflict of Interest Statement

The authors declare that the research was conducted in the absence of any commercial or financial relationships that could be construed as a potential conflict of interest.

## Footnotes

1 https://www.qiagen.com

2 http://www.ncbi.nlm.nih.gov/geo/info/linking.html

3 www.rproject.org

4 http://www.silkscientific.com

## Abbreviations

ALS: Amyotrophic Lateral Sclerosis
ARC: activity-regulated cytoskeleton-associated protein
BDNF: brain derived neurotrophic factor
CORT: corticosterone
CPEB3: the cytoplasmic polyadenylation protein
CREB1: cAMP responsive element binding protein 1
CUMS: chronic unpredictable mild stress
DG: dentate gyrus
EPH: EPHRINS
EPHS: EPHRIN receptor
GLUA2: AMPA receptor subunit 2
GRIN: glutamate ionotropic receptor NMDA type subunit 1
HDAC: histone deacetylase
KCC2: K-Cl co-transporter-2
LTP: long-term potentiation
MAPT: microtubule associated protein Tau
MDD: major depressive disorder
MEF2D: myocyte enhancer factor 2D
miRNAs: microRNAs
mTOR: mammalian target of rapamycin
NCOR1: nuclear receptor co-repressor 1
NFAT: nuclear factor of activated T-cells
PI3K: phosphatidylinositol-3-kinase
PTEN: phosphatase and tensin homolog
SEMA: semaphorin
SGK1: serum glucocorticoid dependent kinase 1
SNCA: synuclein alpha
TP53: tumor protein 53.
