# Supplementary material for: MicroRNA Profiling and Bioinformatics Target Analysis in Dorsal Hippocampus of Chronically Stressed Rats: Relevance to Depression Pathophysiology
Source: Front Mol Neurosci. 2018 Aug 6;11:251. doi: 10.3389/fnmol.2018.00251 (PMC6088391; doi:10.3389/fnmol.2018.00251)
Supplement: Supplementary file 5 [file Presentation_1.PDF]

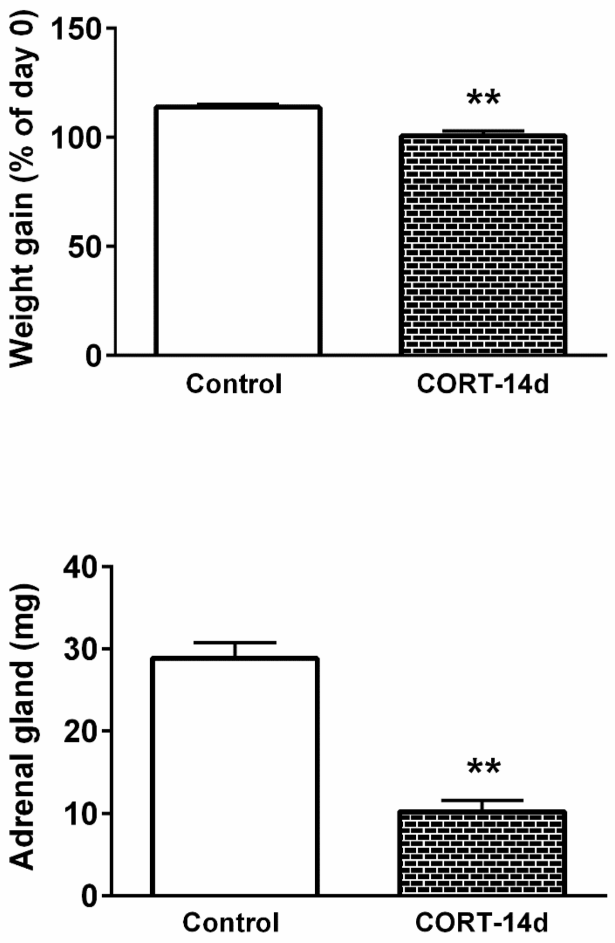

Figure S1. Effect of chronic CORT administration on body weight gain and adrenal weight. Animals were injected s.c. once per day with a 30 mg/kg/day dose of corticosterone during 14 days (CORT-14d, n=5). Control animals (n=5) were injected with a similar volume of vehicle (propylene glycol). Animals were sacrificed 24 h after the last injection. (A) Variation of body weight gain was evaluated at the end point of treatment. All rats were weighed daily during 14 consecutive days. The graph represents the change in body weight as a percentage of the initial weight. (B) Adrenal gland weight of control and CORT-treated animals. Graph represents mean ± S.E.M. Data were analyzed by two-tailed Mann-Whitney test, ** $P < 0.01$.
